# Supplementary material for: Spin skyrmion gaps as signatures of strong-coupling insulators in magic-angle twisted bilayer graphene
Source: Nat Commun. 2023 Oct 21;14:6679. doi: 10.1038/s41467-023-42275-6 (PMC10590429; doi:10.1038/s41467-023-42275-6)
Supplement: Supplementary file 1 — Supplementary Information [file 41467_2023_42275_MOESM1_ESM.pdf]

Supplementary Information for: Spin skyrmion gaps as signatures of strong-coupling insulators in magic-angle twisted bilayer graphene

Jiachen Yu,[1,2,*] Benjamin A. Foutty,[2,3,*] Yves H. Kwan,[4,*] Mark E. Barber,[1,2] Kenji Watanabe,[5] Takashi Taniguchi,[6] Zhi-Xun Shen,[1,2,3,7] Siddharth A. Parameswaran,[4] and Benjamin E. Feldman[2,3,7,†]

[1]*Department of Applied Physics, Stanford University, Stanford, CA 94305, USA*
[2]*Geballe Laboratory for Advanced Materials, Stanford, CA 94305, USA*
[3]*Department of Physics, Stanford University, Stanford, CA 94305, USA*
[4]*Rudolf Peierls Centre for Theoretical Physics, University of Oxford, Oxford OX1 3PU, United Kingdom*
[5]*Research Center for Electronic and Optical Materials, National Institute for Materials Science, 1-1 Namiki, Tsukuba 305-0044, Japan*
[6]*Research Center for Materials Nanoarchitectonics, National Institute for Materials Science, 1-1 Namiki, Tsukuba 305-0044, Japan*
[7]*Stanford Institute for Materials and Energy Sciences, SLAC National Accelerator Laboratory, Menlo Park, CA 94025, USA*

Contents

1. Device Information and Additional Figures 2

2. Twist angle dependence 2

3. Hofstadter-Chern insulators 3

4. Hartree-Fock calculations 4
A. Ground state at $\nu = +2$ 4
B. Skyrmion excitations 5
C. Field dependence of the gap 6

5. Sigma model analysis of spin skyrmions at $|\nu| = 2$ 7
A. Variational estimates for single intra-Chern skyrmions 8
B. Intervalley Hund's coupling 9
1. Ferromagnetic Hund's 9
2. Antiferromagnetic Hund's 10

6. Alternative mechanisms 10
A. Orbital magnetization 10
B. Orbital effects at larger fields 11
C. Comparison to parallel magnetic field 12

7. Evidence of a spontaneous charge neutrality gap 12

8. Chemical potential range 13

References 17

*These authors contributed equally
†Electronic address: bef@stanford.edu

1. DEVICE INFORMATION AND ADDITIONAL FIGURES

Fig. S1 shows an optical micrograph of the magic-angle twisted bilayer graphene (MATBG) device. Areas 1 and 2 in the main text are denoted by blue and red circles, respectively.

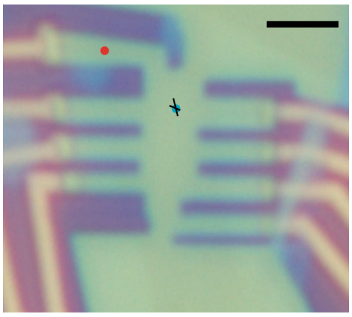

FIG. S1: **Optical micrograph of the device.** Blue and red dots represent Areas 1 and 2, respectively. The linear trajectories in Fig. 1a and Fig. S4a (see below) are denoted by the black lines. Scale bar, 2 um.

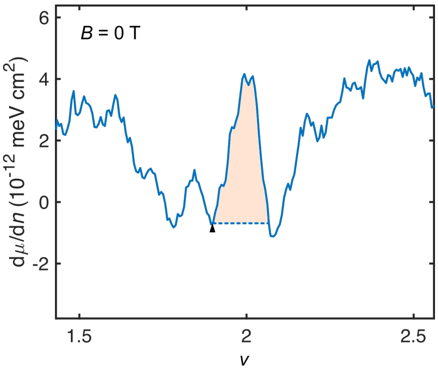

FIG. S2: **Demonstration of gap extraction procedure.** The gap is identified as the area enclosed between the measured $d\mu/dn$ and the horizontal dashed line. The position of the horizontal dashed line is determined by the higher of the two local minima in $d\mu/dn$ surrounding the $\nu = 2$ incompressible peak. The numerically integrated gap is proportional to the area shaded in orange.

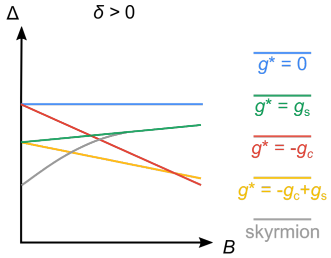

FIG. S3: **Energy gap as a function of B for $\delta > 0$.** The alternative $B = 0$ energy band offset leads to different Δ_2 evolution at high fields, but the low-field crossover from skyrmion gap to particle-hole gap remains qualitatively similar to Fig. 2g.

2. TWIST ANGLE DEPENDENCE

We comment on the twist angle dependence of the strong-coupling insulators in more detail. In this device, we did not detect zero-field gaps in Area 2 where the twist angle is closer to 1.06°. Even in Area 1, the insulating gap is present in a limited region (Fig. S4). Figure S4a shows another spatial line cut in Area 1 in addition to that

from Fig. 1a. Correlated insulators at $\nu = \pm 2$ are again visible in certain locations (green and blue highlighting, respectively, in Fig. S4b). However, the extracted gaps do not always match, even for similar twist angles (Fig. S4c). The above observations indicate that the stability of the strong-coupling insulators are sensitive to details and further highlight the importance of local measurements. While we did not observe non-monotonic gaps at lower angles experimentally, we comment that numerically, skyrmions can be stabilized near 1.06° as well. Thus, we expect that the non-monotonicity in gap dependence could also arise at smaller angles if not for other factors such as strain.

Given the above nuance, we do not anticipate that non-monotonic B dependence is universal. Our work nevertheless demonstrates that skyrmion quasiparticles are sometimes preferred. For the same reasons, the absence in our data of non-monotonic B dependence at smaller angles does not necessarily suggest that the skyrmions are only stabilized when θ is larger. Rather, it is in line with a central point revealed by our local measurements and numerical analysis: multiple microscopic parameters including but not limited to twist angle contribute to determining the properties of the correlated ground states and their charged excitations.

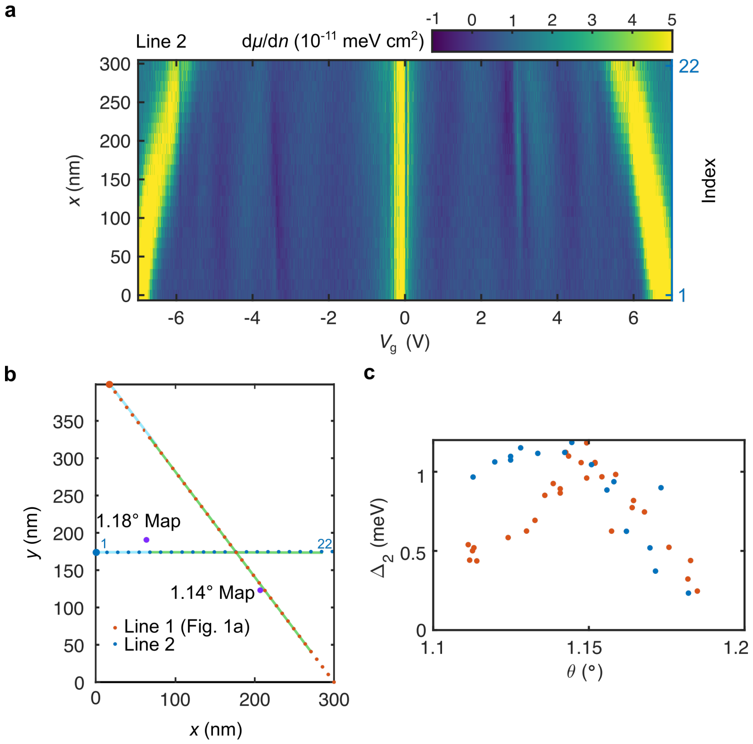

FIG. S4: **a**, $d\mu/dn$ as a function of gate voltage V_g along an additional linear trajectory. **b**, Real-space position of the two linear trajectories in Fig. 1a and Fig. S3a. The $x = 0$ locations in each line are denoted by larger dots. Green (blue) highlighting indicates locations where $\nu = 2(-2)$ gaps are visible at $B = 0$. Spatial locations of the field sweeps in Fig. 2a,b are labeled by purple dots. **c**, Δ_2 as a function of θ, extracted from both linear trajectories.

3. HOFSTADTER-CHERN INSULATORS

Figure S5 shows the Chern insulators/zeroth Landau levels (zLLs, defined as all Landau levels emanating from $\nu = 0$) observed in Fig. 2a. These gapped states can be defined by two quantum numbers, (t, s), following the Diophantine equation: $\nu = t\frac{\phi}{\phi_0} + s$, where t represents the Chern number of the gapped state, s is the band-filling index (zero-field intercept), ϕ is the flux per moiré unit cell and ϕ_0 is the magnetic flux quantum.

The broken-symmetry Chern insulators that we observe match those generally found in prior studies [S1–S8] and follow the same pattern as obtained from Area 2 of the same device, which was systematically studied in [S8]. The only exception is that we see a translation symmetry broken Chern insulator $(3, 1/2)$ on the electron-doped side but not its hole-doped counterpart, whereas in [S8] only the latter was seen. A similar pattern of Chern insulators is also reproduced in the $\theta = 1.18°$ location, as is shown in Fig. S6.

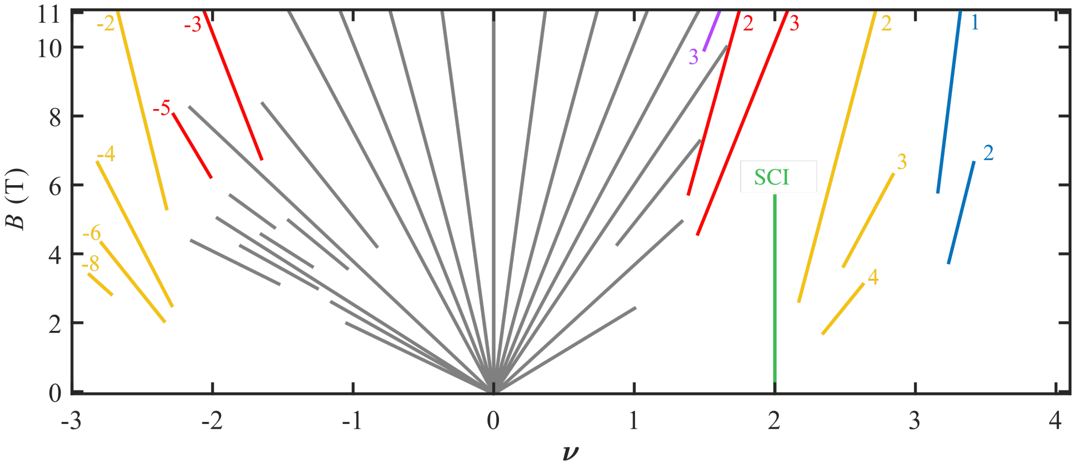

FIG. S5: **Wannier diagram for the $\theta = 1.14°$ location.** Selected incompressible states are labeled by their Chern number. Gray, red, yellow, blue and purple colors correspond to states with different integer and fractional (1/2) intercepts, respectively. The gapped strong-coupling insulator (SCI) at filling factor $\nu = 2$ is highlighted in green.

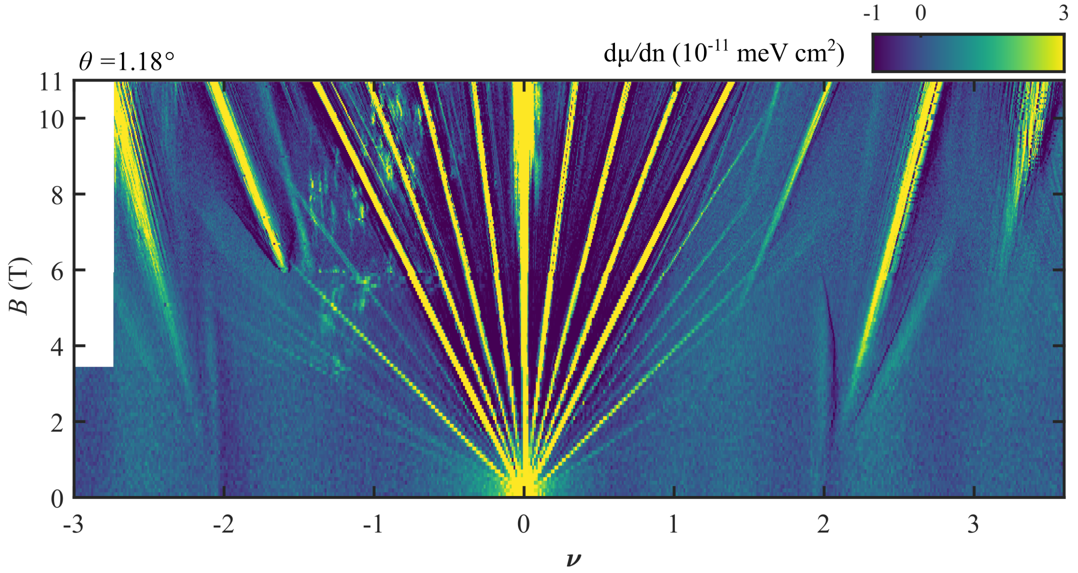

FIG. S6: **Landau fan at a second location.** $d\mu/dn$ as a function of ν and B measured at a second location with twist angle $\theta = 1.18°$ (Fig. 2b,c) showing $\nu = \pm 2$ gapped ground states at low B.

4. HARTREE-FOCK CALCULATIONS

In this section, we provide details of our Hartree-Fock (HF) calculations on the central moiré bands of the interacting Bistritzer-MacDonald (BM) [S9] model near filling factor $|\nu| = 2$. Since we neglect non-local tunneling corrections, the results at $\nu = -2$ and $+2$ are related by the approximate particle-hole symmetry of the model. For concreteness we work at $\nu = +2$. To properly define interactions and maintain the approximate particle-hole symmetry, one needs to introduce a 'subtraction scheme', which prevents double-counting of interactions [S10, S11]. We choose the 'average scheme', which subtracts off a density matrix $P_0 = \frac{1}{2}I$ in the central bands. For the interlayer hopping parameters, we fix $w_{\rm AB} = 110\,\text{meV}$, but allow $w_{\rm AA}$ to vary.

A. Ground state at $\nu = +2$

At $\nu = +2$, the ground state in HF is a Kramers intervalley coherent (KIVC) insulator which breaks spinless time-reversal symmetry (TRS) $\mathcal{T} = \tau_x\mathcal{K}$ but is invariant under $\mathcal{T}' = \tau_y\mathcal{K}$, where τ_i are Pauli matrices in valley space and

$\mathcal{K}$ is complex conjugation. A representative member of these states is approximated well by the spin-polarized density matrix $P \propto (1+s_z)\tau_x\sigma_y + (1-s_z)\tau_0\sigma_0$, where s_i and σ_i act in spin and sublattice (Chern band) space respectively. The Chern basis refers to the basis which diagonalizes the microscopic sublattice operator σ_z, and separates into Chern sectors with $C = \tau_z\sigma_z$. In the absence of intervalley Hund's coupling, there is a degenerate manifold of states generated by independent spin rotations in each valley [$SU(2)_K \times SU(2)_{K'}$ symmetry], and valley rotations [$U(1)_V$ symmetry]. The KIVC has Chern number $C = 0$, consistent with the low-field vertical feature in $d\mu/dn$ observed in the $\nu - B$ plane at $|\nu| = 2$. It has been predicted that the KIVC gives way to a spin-unpolarized gapped incommensurate Kekulé spiral (IKS) [S12], also with $C = 0$, at moderate heterostrain. If the sample is hBN-aligned, a sufficiently strong sublattice coupling is theoretically expected to stabilize a sublattice-polarized valley Hall (VH) insulator, though even without sublattice coupling, the VH is closely competitive with the KIVC. More generally, the KIVC and VH states are part of a broader family of low-lying 'strong-coupling states' which share the same topological properties. Hence at $B = 0$, there are two classes of candidate insulators with trivial Chern number: the IKS and the strong-coupling states.

For the rest of this section, with the aim of understanding the non-monotonic gap dependence at $\nu = +2$, we investigate the excitations of the KIVC. We focus on this state because there is no evidence of hBN alignment, which would lead to a quantized anomalous Hall state at $\nu = +3$ (which we do not observe), and a large zero-field $\nu = 0$ single-particle gap of size $\gtrsim 10$ meV [S13, S14] (much greater than we observe experimentally). However, we note that we expect the excitations of other strong-coupling insulators to behave in a qualitatively similar fashion. The IKS only exhibits a decreasing gap with increasing magnetic field, inconsistent with our observations in the 1.18° region.

B. Skyrmion excitations

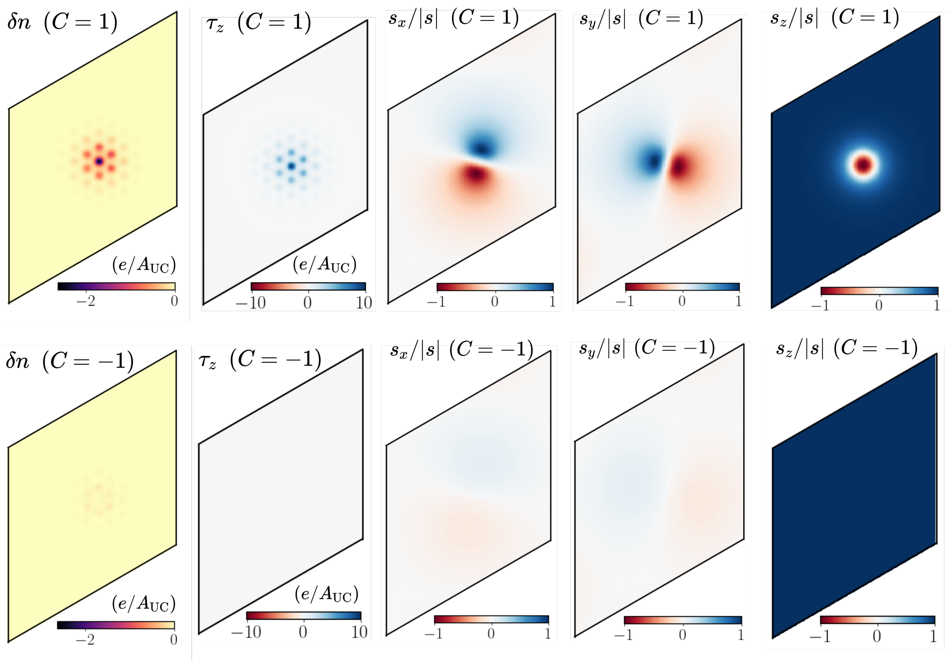

FIG. S7: **Spatial profile of hole skyrmions of the $\nu = +2$ KIVC insulator. Top:** Charge density in the $C = 1$ Chern sector of the hole skyrmion δn, measured relative to that of the KIVC insulator at $\nu = +2$. The dotted pattern arises from the charge localization of the central band wave functions to the moiré AA-stacking regions. Also shown is the valley polarization τ_z and the spin profile. **Bottom:** Same except for the $C = -1$ sector. System size is 13×13, $\theta = 1.15°$, $w_{\rm AA} = 30\,$meV, relative permittivity $\epsilon_r = 6$.

The incompressible peak at integer filling is a probe of the lowest-energy charged excitations of the insulator through the relation

$$\Delta_\nu = E[N_\nu + 1] + E[N_\nu - 1] - 2E[N_\nu], \qquad (1)$$

where $E[N]$ is the ground state energy at particle number N, and N_ν denotes the particle number at filling ν. We estimate $\Delta_{\nu=+2}$ in our self-consistent numerics at different N. If translation symmetry is enforced, then HF is only

able to capture particle-like excitations of the insulator. However, relaxing this constraint permits the investigation of more general charged excitations, e.g. skyrmions, which may have a lower energy. We do not find this for a single added electron, which is consistent with the general pattern of greater interaction-renormalized band dispersion upon doping the correlated insulators away from charge neutrality [S15]. On the other hand, as shown in Fig. S7 (see also Fig. 3 in the main text), the lowest-energy hole excitation has non-trivial spatial modulation. The approximate particle-hole symmetry about neutrality means that these trends are reversed at $\nu = -2$, with skyrmions appearing for doping towards neutrality and absent for doping away from neutrality. Resolving the charge density, relative to the spin-polarized parent insulator, in spin, valley, and Chern space reveals that the added hole predominantly enters as a spin skyrmion in one Chern sector (left column of Fig. S7). This is verified by computing the spin Pontryagin density. The skyrmion core also attains near-complete valley polarization in the same Chern sector, but there is no evidence of a non-trivial winding in pseudospin space. The other Chern sector does not change significantly. Qualitatively similar behavior is found for different twist angles, w_{AA}, and interaction subtraction schemes.

In contrast to a skyrmion in a system with perfect $SU(2)$ symmetry, the skyrmion in Fig. S7 is finite in size, which can be confirmed by changing the simulation size. Increasing w_{AA} also shrinks the skyrmion and reduces its relative energy gain, suggesting that finite w_{AA} introduces an anisotropy term to the relevant Goldstone manifold. For the parameters studied, we are able to obtain skyrmions as the lowest-energy excitations up to $w_{\mathrm{AA}} = 40\,\mathrm{meV}$. We believe that mean-field theory likely underestimates the stability of skyrmions, relative to particle-like excitations, for two reasons. First it seems natural that the skyrmion, being a non-trivial correlated object with soft modes from spatial texturing, can benefit more from beyond mean-field fluctuations. In the regime of small skyrmion size at finite fields, a calculation that captures the dressing and quantum fluctuations of such excitations is possible [S16, S17]. Second, our calculation produces localized skyrmions, but one can lower the energy further by restoring the symmetry and producing 'plane-wave' skyrmions [S15]. Ultimately, we view our numerical calculations as a proof-of-principle demonstration of the feasibility of spin skyrmions in the KIVC state in TBG, which captures the main features of the physics. We note that the value of the chiral ratio $\kappa = w_{\mathrm{AA}}/w_{\mathrm{AB}}$ is not precisely known, and estimates vary in the range $0.5 - 0.8$ [S18]. It has been proposed that κ can be renormalized downwards due to the remote bands [S19]. A lower chiral ratio is generally beneficial for skyrmions owing to reduced band dispersion, closer proximity to the $U(4)_K \times U(4)_{K'}$-symmetric limit, and reduced Berry curvature modulation.

C. Field dependence of the gap

The skyrmion in Fig. S7 involves ~ 10 spin flips. The large number of spin flips suggests that Zeeman coupling to the external field will be substantial. In Fig. 3 of the main text, we plot the dependence of Δ_2 on an externally applied Zeeman field (blue squares). Note that mean-field theory is generally expected to overestimate band gaps. At zero field, Δ_2 is smaller than the particle-hole gap Δ_{ph} (dashed line) because of the reduced energy of hole skyrmions. Δ_2 rises at small fields, and crosses over to the particle-hole band gap at large fields. This is consistent with the fact that the skyrmion shrinks in size to minimize the Zeeman energy, thereby paying an increased Coulomb cost. This is corroborated by the reduction in the degradation of the spin moment in HF. Note that the increase of Δ_2 at $B = 0$ is non-singular, and has positive second derivative. The fact that Δ_{ph} is constant implies that the HF bands immediately below and above the Fermi energy are of the same spin.

The perpendicular external magnetic field is expected to couple to the orbital degrees of freedom as well. A full microscopic treatment of this is difficult, especially in multiband moiré systems. For sufficiently weak fields below the Hofstadter regime, we choose to model the orbital coupling as an effective field that couples to opposite Chern sectors with effective g-factor $g_c > g_s$. This term is odd under spinless time reversal, and linearly closes Δ_{ph}, and hence Δ_2 at high fields (red line in Fig. 2g, Fig. S3 and Fig. 3c). The skyrmion excitation is not affected since it primarily involves only a single Chern sector. The net result is a non-monotonic gap dependence, where the crossover to the particle-hole gap occurs at a somewhat smaller B-field than in the absence of orbital effects. The quantitative value of the crossover field is detail-dependent, such as the chiral ratio and the effective dielectric constant. For instance, we expect that a reduced chiral ratio will increase the energetic gain of skyrmions due to the reduction of the λ anisotropy. Similarly, increased interaction strength will benefit skyrmions. These will act to increase the crossover field.

While we see no evidence for hBN-alignment in experiment, we summarize the theoretical expectations for the skyrmion physics in this regime for completeness. In the presence of substrate alignment, a large extrinsic sublattice gap opens at charge neutrality leading to energetically resolved Chern bands at the single-particle level. Here the only $C = 0$ strong-coupling insulator is the valley Hall (VH) state which is valley-diagonal and involves sublattice polarization in each valley. As proposed in [S20], doped charges can enter as spin skyrmions by exploiting the Chern topology of the substrate-split bands, and the $SU(2)_K \times SU(2)_{K'}$ spin-rotation symmetry in the absence of Hund's coupling. Variational calculations based on a non-linear sigma model (NLSM) predict a low-field singular increase

of the gap $\Delta \sim B^{\alpha}$, with α between 1/2 and 1/3 depending on the screening properties [S20]. For large fields when the skyrmion becomes unfavorable, the particle-hole gap closes linearly due to the valley Zeeman coupling, with theoretical estimates of $g_v \simeq 2-6$ [S21]. In the presence of ferromagnetic Hund's coupling, which reduces the spin symmetry to $SU(2)$, the singular behavior of the skyrmion gap at $B=0$ is cut off since the other valley effectively imposes a constant Zeeman field. However, $d^2\Delta/dB^2$ remains negative. For antiferromagnetic Hund's coupling, the commensurate insulator is a spin-valley locked state, whose valley moments cant in the presence of an external field. The skyrmion gap is constant until the critical field B_c where the valley moments of the insulator align. Above this, Δ_2 increases until it switches over to the decreasing particle-hole gap. B_c depends sensitively on the relative strengths of intervalley Coulomb scattering and phonon scattering, but has been estimated to be in the range $0.1-2\,\mathrm{T}$. Finally, we comment on the quantitative discrepancy between the experimentally measured and numerically evaluated gap sizes. A likely effect that suppresses the gap is fluctuations beyond mean-field, and we expect this to be the dominant mechanism as well as monotonic in B. Intuitively, we anticipate the magnitude of fluctuations to depend roughly on the size of the gap itself. While this could alter the scale of the non-monotonicity, we find it unlikely that it could itself seed non-monotonicity. On the other hand, the Zeeman suppression of the spin skyrmion and its competition with single-particle excitations is a transparent physical principle that we expect to be robust beyond the mean-field regime. In conclusion, this quantitative discrepancy does not affect the validity of the theoretical approach adopted in the main text nor the robustness of the results.

5. SIGMA MODEL ANALYSIS OF SPIN SKYRMIONS AT $|\nu|=2$

While HF already provides explicit skyrmion solutions of the KIVC in a microscopic model, the NLSM gives a complementary field-theoretic perspective that is a good approximation in the limit of large skyrmions, narrow bandwidth, and small deviations from the chiral limit $\kappa=0$. Therefore, we cover the NLSM in this section for completeness. The NLSM is a continuum description that captures smooth spatial variations in the correlated state in MATBG [S22, S23]. The NLSM coarse grains real-space modulations on the moiré scale, as well as momentum-space features of the dispersion and Bloch functions. By focusing on the (approximate) continuous symmetries of the problem, the NLSM facilitates the analysis of the the possible skyrmion excitations in the system, and the identification of the primary factors that affect their stability. We start by introducing the energy functional of the NLSM, whose terms stabilize the KIVC at $|\nu|=2$. First ignoring the intervalley Hund's coupling, we study the singly-charged skyrmions of the KIVC, and show that spin skyrmions are favored over pseudospin skyrmions. Then, we discuss the effects of the Hund's coupling. We emphasize that the NLSM also captures the analogous physics of other strong coupling insulators, which simply correspond to different uniform directions in the sigma model. These alternatives can be stabilized by changing the signs of the anisotropy terms. For concreteness, we base the following discussion on the KIVC state to model the HF results.

The correlated state in MATBG is parameterized by Hermitian 4×4 matrices Q^C, one for each Chern sector C. We define $Q=\mathrm{diag}(Q^+,Q^-)$, which satisfies $Q^2=1$ and $\mathrm{tr}Q=2\nu$. Hence, we are considering states which do not involve inter-Chern coherence. In Chern sector $C=+1$, Q^+ acts on the Chern basis components $\{KA\uparrow, KA\downarrow, K'B\uparrow, K'B\downarrow\}$, while Q^- in $C=-1$ acts on $\{KB\uparrow, KB\downarrow, K'A\uparrow, K'A\downarrow\}$. The eigenvectors of Q^C with eigenvalue $+1$ (-1) correspond to filled (empty) bands in Chern sector C.

The leading parts of the NLSM energy functional consist of the gradient term $\sim\int d\mathbf{r}\,\mathrm{tr}(\nabla Q)^2$, which prefers to smooth out spatial modulations in Q, and a long-range Coulomb interaction piece. The latter term is of the form $\int d\boldsymbol{r}d\boldsymbol{r'}\delta\rho(\boldsymbol{r})V(\boldsymbol{r})\delta\rho(\boldsymbol{r'})$, where $V(\boldsymbol{r})$ is the (gate-screened) Coulomb potential, and $\delta\rho(\boldsymbol{r})$ can be non-zero due to the link between the topological density and the charge density in Chern bands. Both of the above terms satisfy a large $U(4)\times U(4)$ symmetry corresponding to independent rotations within each Chern sector.

Consider now the leading anisotropic parts of the NLSM energy density

$$\mathcal{E}_{\mathrm{anis}}[Q]=\frac{J}{4}\mathrm{tr}(Q\gamma_x)^2-\frac{\lambda}{4}\mathrm{tr}(Q\gamma_x\eta_z)^2-g_B\mathrm{tr}(Qs_z) \tag{2}$$

$$\gamma_{x,y,z}=(\sigma_x,\sigma_y\tau_z,\sigma_z\tau_z),\qquad \eta_{x,y,z}=(\sigma_x\tau_x,\sigma_x\tau_y,\tau_z). \tag{3}$$

The J-term arises from second-order perturbation theory in the inter-Chern part of the dispersion. The λ-term is first-order in the interaction and quadratic in the intersublattice component of the form factor, which vanishes in the chiral limit $\kappa=0$. These two terms reduce the symmetry to $SU(2)_K\times SU(2)_{K'}$ corresponding to independent spin rotations within each valley. The g_B term is proportional to the spin Zeeman splitting, and we have ignored the Hund's coupling.

For notational convenience, we consider $\nu=-2$ such that one band in each Chern sector is filled. A convenient parameterization of Q is in terms of two $\mathbb{CP}^3$ fields $Z^C=[v^C_{K\uparrow},v^C_{K\downarrow},v^C_{K'\uparrow},v^C_{K'\downarrow}]^T\sim|v^C\rangle$, where the sublattice band

index is implicit from the Chern and valley number. $|v^C\rangle$ represents the filled state in each Chern sector. We have $Q^C_{\alpha\beta} = 2v^C_\alpha(v^C_\beta)^* - \delta_{\alpha\beta}$, and the energy density becomes

$$\mathcal{E}_{\text{anis}}[v^+, v^-] = 2J|\langle v^+|v^-\rangle|^2 - 2\lambda|\langle v^+|\eta_z|v^-\rangle|^2 - g_B\left(\langle v^+|s_z|v^+\rangle + \langle v^-|s_z|v^-\rangle\right). \tag{4}$$

If we keep v^- fixed, then from the perspective of v^+, there are three types of anisotropic fields acting on it. The J-term acts to ensure that v^+ remains orthogonal to v^- (whether that be by having an orthogonal spin or pseudospin alignment, or a mixture of both). Hence there is a large subspace in which the J-term remains satisfied. On the other hand, the λ-term favors v^+ being aligned with $\eta_z v^-$, meaning that there is only one unique point in the $C = 1$ $\mathbb{CP}^3$ manifold that maximally satisfies this term. The g_B-term aligns the $\mathbb{CP}^3$ fields along the Zeeman axis. $\mathcal{E}_{\text{anis}}[v^+, v^-]$ is minimized by the spin-polarized KIVC, where the uniform fields v^+, v^- are spin-polarized along z, and pseudospin-polarized in-plane along opposite directions. Changing the signs of the coefficients J, λ stabilizes other strong-coupling orders. For example, a negative value of λ would instead favour the spin-polarized VH, where v^+, v^- are pseudospin-polarized in opposite directions along the poles.

A. Variational estimates for single intra-Chern skyrmions

Consider a singly-doped system where a skyrmion has entered the $C = +1$ sector. At infinity, the $r \to \infty$ configuration consists of $Z^+_\infty = \frac{1}{\sqrt{2}}[1,0,1,0]^T$ and $Z^-_\infty = \frac{1}{\sqrt{2}}[1,0,-1,0]^T$, as expected for a KIVC insulator. The skyrmion is parameterized by its $C = +1$ core configuration Z^+_0 at $r = 0$, satisfying $(Z^+_\infty)^\dagger Z^+_0 = 0$. We assume that Z^- remains unchanged for all space. The skyrmion is described by

$$Z^+_{\text{skyr}}(\boldsymbol{r}) = \frac{(x+iy)Z^+_\infty - \lambda(r)Z^+_0}{\sqrt{r^2 + \lambda(r)^2}}. \tag{5}$$

Generally, Z^+_0 can be described by four real angles in the following way [S24]

$$Z^+_0 = \cos\frac{\alpha}{2}\begin{pmatrix}\frac{1}{\sqrt{2}}\\ 0\\ -\frac{1}{\sqrt{2}}\\ 0\end{pmatrix} + \sin\frac{\alpha}{2}e^{i\beta}\begin{pmatrix}0\\ \cos\frac{\theta_p}{2}\\ 0\\ \sin\frac{\theta_p}{2}e^{i\phi_p}\end{pmatrix} \to |v^+(\theta,\phi)\rangle = \begin{pmatrix}\frac{1}{\sqrt{2}}\left(\cos\frac{\theta}{2} + \cos\frac{\alpha}{2}\sin\frac{\theta}{2}e^{i\phi}\right)\\ \sin\frac{\alpha}{2}\sin\frac{\theta}{2}\cos\frac{\theta_p}{2}e^{i(\phi+\beta)}\\ \frac{1}{\sqrt{2}}\left(\cos\frac{\theta}{2} - \cos\frac{\alpha}{2}\sin\frac{\theta}{2}e^{i\phi}\right)\\ \sin\frac{\alpha}{2}\sin\frac{\theta}{2}\sin\frac{\theta_p}{2}e^{i(\phi+\phi_p+\beta)}\end{pmatrix}. \tag{6}$$

α, β describe whether the skyrmion is a pure pseudospin rotation or has some spin rotation. θ_p, ϕ_p parameterize the pseudospin orientation in the case of a spin rotation. θ, ϕ parameterize the coordinates of the skyrmion. This ansatz leads to the energy density

$$\mathcal{E}_{\text{anis}}(\theta,\phi) = -\lambda(1+\cos\theta) + J\frac{1+\cos\alpha}{2}(1-\cos\theta) - g_B\left(\frac{1+\cos\alpha}{2} + \frac{1-\cos\alpha}{2}\cos\theta\right) \tag{7}$$

$$= -\lambda + \frac{J-g_B}{2} + \frac{J-g_B}{2}\cos\alpha - \left[\lambda + \frac{J+g_B}{2} + \frac{J-g_B}{2}\cos\alpha\right]\cos\theta \tag{8}$$

$$\equiv -\lambda + \frac{J-g_B}{2} + \frac{J-g_B}{2}\cos\alpha - g(\alpha)\cos\theta. \tag{9}$$

For pure pseudospin skyrmions ($\alpha = 0$), the skyrmion sees an effective field of strength $\lambda + J$. For spin skyrmions ($\alpha = \pi$), the skyrmion sees an effective field of strength $\lambda + g_B$. No matter the rotation direction, the skyrmion experiences the field due to λ. We expect spin skyrmions to be energetically favored over pseudospin skyrmions. Note that the spin skyrmion has an additional degeneracy since the energy density is independent of θ_p, ϕ_p. This will be lifted by subleading terms not included in the NLSM, such as those arising from the intra-Chern dispersion [S25]. The net result is that the skyrmion core becomes valley polarized with $\theta_p = 0, \pi$, which is consistent with the HF results. We will not consider this term (which we assign a characteristic energy scale h_A to) in the following.

We can use a simple scaling argument, which will not capture logarithmic corrections, to obtain a rough estimate of the skyrmion radius R and energy E_{skyr}. By dimensional analysis, we approximate the total skyrmion energy

$$E_{\text{skyr}}(R,\alpha) = \frac{e^2}{R} + g(\alpha)\left(\frac{R}{a_M}\right)^2 + \frac{J-g_B}{2}\cos\alpha - \lambda + \frac{J-g_B}{2}, \tag{10}$$

which now includes the long-range interaction $\sim e^2$. The gradient term is R-independent and hence omitted. We optimize this by finding the ideal radius R in $E_{\rm skyr}$ for every α, and then minimizing over possible values of α. We find a spin ($\alpha=\pi$) and pseudospin ($\alpha=0$) skyrmion for $J>g_B$ and $J<g_B$ respectively, with

$$J>g_B \to (\alpha=\pi);\quad E_{\rm skyr}=\frac{3e^2}{2R}-\lambda;\quad R=\left(\frac{e^2a_M^2}{2(\lambda+g_B)}\right)^{1/3} \tag{11}$$

$$J<g_B \to (\alpha=0);\quad E_{\rm skyr}=\frac{3e^2}{2R}-\lambda+J-g_B;\quad R=\left(\frac{e^2a_M^2}{2(\lambda+J)}\right)^{1/3}. \tag{12}$$

Note the presence of λ which cuts off any singularity as $B\to 0$.

B. Intervalley Hund's coupling

Intervalley Hund's coupling J_H breaks the set of spin symmetries down to $SU(2)_S$, and manifests as an exchange interaction between the spins in the two valleys. This will resolve the spin degeneracy to be spin ferromagnetic ($J_H<0$) or spin-valley-locked with no net spin moment ($J_H>0$). Theoretically, J_H is predicted to have opposing contributions from intervalley Coulomb scattering and phonon scattering, such that the overall sign is uncertain [S20]. Experimental studies of the transport and magnetization response to in-plane magnetic fields have yielded mixed conclusions for the spin polarization at $\nu=2$ [S2, S7, S26–S28].

The Hamiltonian density for a uniform state is parameterized as [S20]

$$h_{\rm IVH}=J_H\boldsymbol{S}_K\cdot\boldsymbol{S}_{K'} \tag{13}$$

$$=J_H\sum_i\left(\sum_{\sigma s_1 s_2}c^\dagger_{K\sigma s_1}s^i_{s_1s_2}c_{K\sigma s_2}\right)\left(\sum_{\sigma' s_3 s_4}c^\dagger_{K'\sigma' s_3}s^i_{s_3s_4}c_{K'\sigma' s_4}\right). \tag{14}$$

Its contribution can be added to the anisotropic NLSM energy density

$$\mathcal{E}_{\rm anis}[Q]=\frac{J}{4}{\rm tr}(Q\gamma_x)^2-\frac{\lambda}{4}{\rm tr}(Q\gamma_x\eta_z)^2-g_B{\rm tr}(Qs_z) \tag{15}$$

$$+\frac{J_H}{4}\sum_i{\rm Tr}\left[\left(\frac{1+\tau_z}{2}\right)s^iQ\right]{\rm Tr}\left[\left(\frac{1-\tau_z}{2}\right)s^iQ\right]-\frac{J_H}{4}{\rm Tr}\left[\left(\frac{1+\tau_z}{2}\right)s^iQ\left(\frac{1-\tau_z}{2}\right)s^iQ\right] \tag{16}$$

$$\mathcal{E}_{\rm anis}[v^+,v^-]=2J|\langle v^+|v^-\rangle|^2-2\lambda|\langle v^+|\eta_z|v^-\rangle|^2-g_B\left(\langle v^+|s_z|v^+\rangle+\langle v^-|s_z|v^-\rangle\right) \tag{17}$$

$$+J_H\left(\langle v^+_K|\boldsymbol{s}|v^+_K\rangle\cdot\langle v^-_{K'}|\boldsymbol{s}|v^-_{K'}\rangle+\langle v^+_{K'}|\boldsymbol{s}|v^+_{K'}\rangle\cdot\langle v^-_K|\boldsymbol{s}|v^-_K\rangle\right) \tag{18}$$

where the second equality assumes a $\nu=-2$ state with one filled band in each Chern sector, and $|v^C_\tau\rangle$ refers to the valley-τ components of the Chern spinor. We will be interested in the regime $J\gtrsim\lambda\gg g_B,J_H$. We neglect the energy scale h_A associated with terms that resolve the order parameter direction for a single Chern state (e.g. at $\nu=-3$).

1. *Ferromagnetic Hund's*

For $J_H<0$, both Chern states should have their spins aligned—without loss of generality, we align them along the s_z direction. If we assume that the $C=-1$ sector is frozen as $|v^-\rangle=\frac{1}{\sqrt{2}}[1,0,-1,0]^T$, then this acts as an extra Zeeman coupling of strength $-\frac{J_H}{2}$ on the $C=+1$ sector.

2. *Antiferromagnetic Hund's*

For $J_H > 0$, the spins in the two valleys are now anti-aligned. As verified numerically, the energy is minimized in a finite field when the spins are canted relative to the external Zeeman axis

$$|v^+\rangle = \frac{1}{\sqrt{2}}\begin{pmatrix} \cos\frac{\theta_s}{2} \\ \sin\frac{\theta_s}{2} \\ \cos\frac{\theta_s}{2} \\ -\sin\frac{\theta_s}{2} \end{pmatrix} \qquad |v^-\rangle = \frac{1}{\sqrt{2}}\begin{pmatrix} \cos\frac{\theta_s}{2} \\ \sin\frac{\theta_s}{2} \\ -\cos\frac{\theta_s}{2} \\ \sin\frac{\theta_s}{2} \end{pmatrix} \tag{19}$$

where $\cos\theta_s = g_B/J_H$. For $g_B > J_H$, the spins align along the Zeeman field. For a singly-charged $C = +1$ skyrmion, if we assume that the $C = -1$ sector is frozen as above, then the combined effect of the external Zeeman and Hund's coupling is to act as an effective Zeeman coupling of strength $J_H/2$ $(g_B - J_H/2)$ for $g_B < J_H$ $(g_B > J_H)$. Note that for $g_B < J_H$, the effective fields are canted oppositely in the two valleys. The impact on the skyrmion physics for both signs of the Hund's coupling is hence similar to that of the substrate-split VH insulator, except that the λ-term also enters here as a field.

6. ALTERNATIVE MECHANISMS

A. Orbital magnetization

We comment on other candidate explanations for the non-monotonic gap dependence. At weak fields, a magnetic field shifts the levels of the Bloch electrons $\epsilon_k(B) = \epsilon_k(0) - m(k)B$, which defines an effective momentum-local g-factor $g_{\text{orb}}(k)$. This depends on the momentum-resolved orbital magnetization, in particular the contribution due to 'wavepacket self-rotation' [S29, S30]

$$m(k) = \frac{e}{2\hbar}\frac{1}{(2\pi)^2}\text{Im}\langle\partial_k u_k|\times(H_k - \epsilon_k)|\,\partial_k u_k\rangle. \tag{20}$$

At $B = 0$, this vanishes for the IKS insulator since it satisfies $\hat{C}_{2z}$ and time reversal symmetry, TRS (or valley-rotated versions thereof). In particular, no issues arise with band crossings because the relevant bands are distinguished by the spin quantum number. This implies that the IKS, which must be spin-singlet at $\nu = +2$, can only be associated with a strictly decreasing $\Delta_2(B)$, and is hence ruled out in regions of the sample with a non-monotonic gap. If there is a small amount of $\hat{C}_{2z}$-breaking (for instance a small sublattice potential from the substrate that is not strong enough to open a large gap at neutrality), then a non-zero $m(k)$ is allowed, but the magnetization profile has to satisfy TRS. This does not change our conclusions for the gap dependence of the IKS.

Though many strong-coupling candidate states also satisfy valley-rotated versions of $\hat{C}_{2z}$ and TRS, the situation is complicated by the band crossings at time-reversal invariant momenta (TRIM). We first focus on momentum regions away from the TRIM. In this case, one can consider a possible non-skyrmionic scenario for a non-monotonic $\Delta_2(B)$, which relies on the detailed structure of $m(k)$ and a small amount of $\hat{C}_{2z}$-breaking. This also applies to strong-coupling insulators such as the VH state which already break $\hat{C}_{2z}$. Ignoring the intervalley Hund's coupling, the spin Zeeman effect will act to increase the correlated gap with $g_s = 2$. The (indirect) gap is controlled by electron excitations at Γ_{M}, and hole excitations at possibly some other point. Let us focus on the hole excitations for concreteness, since the lowest-energy hole could be away from a TRIM. It could happen that the lowest energy holes have $|g_{\text{orb}}(k)| < 2$, in which case the gap still increases with B, even accounting for the presence of both signs of g_{orb} due to TRS. However, there could be another momentum region whose holes are only slightly higher in energy, but have $|g_{\text{orb}}(k)| > 2$. Hence if we assume we remain in the linear-B regime and $g_{\text{orb}}(k)$ remains fixed even as the band structure deforms, then at some finite field, the relevant lowest-energy holes could swap. In the situation outlined above, the gap would then proceed to close, leading to a non-monotonic gap without skyrmions. We find this explanation unlikely as it requires fine-tuning of the band structure, in contrast with the more generic picture afforded by the skyrmion-gap mechanism. This non-skyrmionic possibility is further ruled out by the behavior of the lowest energy electron excitations at Γ_{M}. There, the bands are degenerate even with $\hat{C}_{2z}$-breaking, and can be directly resolved into Chern states with opposite Chern number. They are then split linearly under the magnetic field with g-factor g_c, which already acts to close the particle-hole gap at $B = 0$.

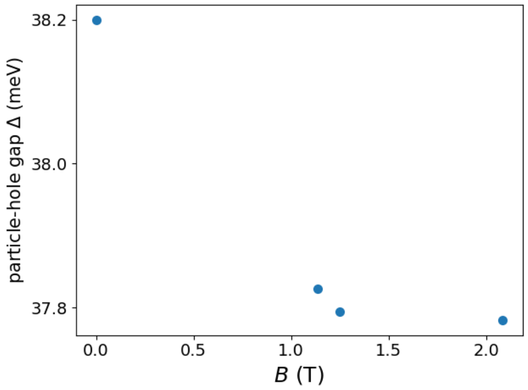

FIG. S8: **Particle-hole gap of the $\nu = 2$ valley Hall insulator as a function of orbital magnetic field.** $w_{\mathrm{AA}} = 30$ meV, $\epsilon_r = 6$, $\theta = 1.15$, gate-screening length (equivalent to hBN thickness) $d_{\mathrm{sc}} = 20\,\mathrm{nm}$, sublattice mass $\Delta = 20\,\mathrm{meV}$.

B. Orbital effects at larger fields

Given the large moiré unit cell of MATBG, the flux quantum $\Phi_0 \sim 25\,\mathrm{T}$ is relatively low, such that the orbital effects of an externally applied field of a few Tesla may play a significant role in altering the properties of the zero-field band structure. Here we discuss the implications of such effects on the spin skyrmion scenario outlined in the previous sections.

Upon approaching the Hofstadter regime, the form factors and band geometric properties of the central bands may change significantly. Ref. [S31] computed various properties of the substrate-split moiré Hofstadter bands as a function of flux and chiral ratio κ, and found that the quantum geometry became increasingly 'ideal' for large fields. In particular, the B-field suppresses fluctuations in the Berry curvature and violations of the so-called trace condition, such that the band geometry more closely resembles that of the lowest Landau level or the BM bands close to the chiral limit $\kappa \to 0$. In addition, the bandwidth generally decreases. Since these bring the system closer to the quantum Hall limit, the formation of skyrmions is expected to be favoured under such conditions, further decreasing the energy of hole skyrmions relative to particle-like holes, and hence decreasing the skyrmion gap. This works in opposition to the spin Zeeman field, so that the net effect could be a reduced slope of the linear increase of Δ_2 at small fields. However, Ref. [S31] finds that the change in quantum geometry at low fields is only prominent for $\kappa \gtrsim 0.7$, which justifies the neglect of band geometry renormalization in our HF calculations which assume a smaller value of the chiral ratio.

For strong-coupling insulators such as the KIVC at non-zero integer filling, theoretical calculations generally find that the interaction-renormalized self-consistent dispersions below and above the gap are highly asymmetric. For $\nu > 0$, the electron bands are significantly more dispersive, which explains why our HF calculations are only able to stablize hole skyrmions. This electron-hole asymmetry has been invoked to explain the directionality of the Landau fans and the sawtooth shape in compressibility. The Hartree corrections are such that the electron bands have a prominent dip at Γ_{M}. This suggests an alternative non-skyrmionic possibility for the non-monotonic gap observed in experiment. For low fields, the Landau quantization of the dispersive band minimum at Γ_{M} leads to a rapid increase of Δ_2. Eventually, this rise saturates when the lowest Landau level hits the spectrum arising from high density-of-states momentum regions away from Γ_{M}. Above this, the band evolution is less clear, but the particle-hole gap may close due to the effective g-factor g_c.

We argue that the above picture is insufficient because it neglects the topology of the bands. In particular, the $C = 0$ strong-coupling insulators at $\nu = 2$ have two conduction bands with opposite Chern numbers $C = \pm 1$. Depending on the sign of the external field, these bands will either lose or gain states to their corresponding valence bands according to the Streda formula. For example, in a non-interacting picture, the lowest conduction band Landau level of one of the Chern bands will flow to become the valence band at one flux quantum, while the opposite happens in the other Chern sector. Since the total filling is fixed at $\nu = 2$ to maintain a gapped state, the particle-hole gap may close from $B = 0$ as the spectral flows of the two Chern sectors cross in opposite directions.

This scenario will be complicated in the presence of interactions. While a full self-consistent calculation of the strong-coupling insulators at finite field is beyond the scope of this paper (see [S32]), we perform a simpler calculation to check that particle-hole gap monotonically closes in a reasonable interacting model. We consider a finite sublattice mass of $20\,\mathrm{meV}$ since this allows us to easily construct a good approximation to a $C = 0$ strong-coupling insulator at $\nu = 2$. In particular, the VH state is built by fully filling a subset of the substrate-split BM bands. Following

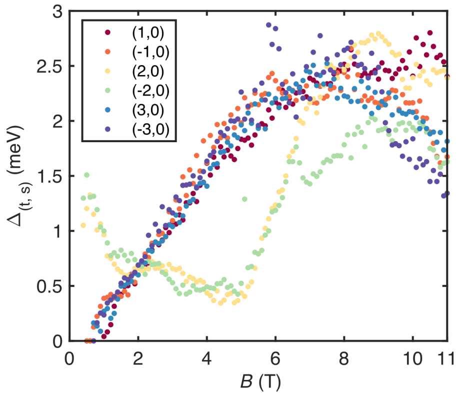

FIG. S9: **Zeroth Landau level (zLL) gaps.** Extracted thermodynamic gaps for broken-symmetry zeroth LLs (zLLs) at the $\theta = 1.14°$ location. States are labeled by (t, s), where t is the Chern number and $s = 0$ is the zero-field intercept.

[S31], this approach can be continued for the Hofstadter bands at finite fields. The field-dependent particle-hole gap is then estimated by calculating the (non-self-consistent) mean-field Hamiltonian, which includes the Hartree and Fock corrections arising from the filled bands. We show representative results in Fig. S8, which demonstrates that the particle-hole gap decreases for small fields. This calculation should incorporate the effects of changes in bandwidth, Berry curvature distribution, and interaction strength, from which we conclude that these effects do not give rise to a non-monotonic gap.

C. Comparison to parallel magnetic field

Magnetic field dependence of the $\nu = 2$ gap has been explored in Ref. [S27] with thermal activation measurement, where a monotonic decrease with $g_{\|} \sim 5\mu_{\mathrm{B}}$ was obtained, quantitatively similar to our measured $g_{\perp}$ in the intermediate field regime. This raises an interesting point of comparison, which we detail below.

To compare $g_{\|}$ and $g_{\perp}$, we first identify factors that may affect $g_{\|}$, which are not limited to spin contribution alone. In addition to the bare spin Zeeman coupling with $g_s \sim 2$, two other mechanisms have been proposed in the literature that would give rise to a more rapid decrease in the gap with parallel field: (1) parallel field breaks C_3 rotational symmetry, which gives rise to a parabolic decrease of the insulating gap for KIVC and VH states [S11]; (2) orbital magnetic moment can also couple to an in-plane magnetic field and give rise to an effective g-factor of order μ_B per moiré unit cell [S33]. Either of these or some combination could potentially explain the large measured $g_{\|}$ in [S27]. Thus, the quantitative similarity between $g_{\|}$ and $g_{\perp}$ does not necessarily imply that orbital effects do not contribute in our experiment. We also comment that the mechanism for spin Zeeman suppression of the skyrmion holds for both parallel and perpendicular fields. Hence, the low-field round-off in Ref. [S27] is also consistent with skyrmionic excitations with a small binding energy.

7. EVIDENCE OF A SPONTANEOUS CHARGE NEUTRALITY GAP

Below we provide a more detailed discussion of the evidence indicating that $\Delta_0(B = 0) \neq 0$, based on its magnetic field dependence, that of the other zLLs, the shape of $\mu(n)$ in the vicinity of the charge neutrality point (CNP), and local conductivity probed with microwave impedance microscopy (MIM).

The magnitude of the gap Δ_0 and its low-field saturation value of 2 meV are remarkably similar in two independent locations (separated by 300 nm) in Area 1 with respective twist angles of $\theta = 1.14°$ and $\theta = 1.18°$. This behavior is consistent with theoritical predictions for a strong-coupling insulator [S32], and it contrasts with that in Area 2 of the same device, where Δ_0 smoothly approaches 0 with decreasing B. As a comparison, we also measure the field dependence of the other zLL gaps near Area 1, shown in Fig. S9. The magnitudes and trends of the zLL gaps with $t \neq 0$ are comparable to those measured in Area 2 [S8]. This contrasts sharply with Δ_0, whose magnitude and trend differs dramatically in the two areas, further highlighting the uniqueness of the behavior of Δ_0.

Another independent metric that we use to assess the presence of a gap is to compare the average slope of $\mu(n)$ in the narrow vicinity of $\nu = 0$, i.e. $\Delta\mu(0) = \mu(0+\epsilon) - \mu(0-\epsilon)$, where $0 < \epsilon \ll 1$ (Fig. S10b,d). The possibility of having a vanishing density of states at $\nu = 0$ from the single-particle band structure (i.e. a Dirac point) complicates a direct

extraction of the thermodynamic gap size, because both will manifest as an incompressible peak with finite width and height in the presence of thermal and doping disorder broadening. This ambiguity naturally raises an alternative potential explanation for the larger $\Delta\mu(0)$, which is that the Dirac velocity in Area 1 is larger than Area 2. However, this hypothesis suffers from two major inconsistencies with the experiment. First, our theoretical calculations indicate that the strain in Area 1 is smaller than that in Area 2. Theoretical calculations [S34, S35] have demonstrated that larger strain leads to significantly larger Dirac velocity at $\nu = 0$. It is therefore inconsistent with a smaller overall $\Delta\mu(0)$ in Area 1, assuming the band structure in Area 1 at $\nu = 0$ retains a massless Dirac spectrum. Secondly, the Bistritzer-MacDonald model predicts a vanishing Dirac velocity at the CNP as θ approaches the magic angle 1.1°, which corresponds to an asymptotically smaller slope near $\nu = 0$ as $\theta \to\sim 1.1°$. While it is known that interactions renormalize the bare band structure drastically, in the vicinity of $\nu = 0$, such effect is minimal [S36]. This implies that for gapless Dirac cones at the CNP, $\Delta\mu(0)$, which scales with the Dirac velocity, should monotonically decrease as $\theta \to\sim 1.1°$. This trend is indeed confirmed in Area 2, where $\mu(n)$ (electron side) can be approximately fitted to a massless Dirac dispersion with a small Dirac velocity of ($< 2 \times 10^5$) m/s. However, in Area 1, the trend is the opposite: as θ decreases towards the magic angle, the slope increases (Fig. S10).

Finally, we present MIM measurements to help determine the gap vs gapless behavior at charge neutrality. MIM measures changes in admittance between a sharp metallic tip and the sample at GHz frequencies. The MIM tip couples capacitively to the sample at these frequencies and by measuring the admittance through reflectometry, a noninvasive local measurement of both capacitive and dissipative responses in the sample can be achieved [S37]. As such, MIM can be used as an independent measure of whether the additional $\nu = 0$ gap gives rise to a more resistive state at the CNP.

The same sample used for the scanning SET measurements was cooled down in a separate cryostat configured for low temperature MIM measurements. The regions studied with SET were located and then characterised as a function of gating with MIM. Fig. S11 shows a comparison of the MIM-Im signal, which changes monotonically with conductivity, as a function of gating in the two regions of the sample, Area 1 and Area 2. The traces are recorded on a grid covering 300 nm × 300 nm over the regions identified with SET. In Area 2, sharp resistive features at the superlattice gaps are observed, and a relatively smaller change in signal occurs at the CNP. This contrasts with the behavior in Area 1, where a clearer and sharp signal is generically observed at the CNP, whereas the superlattice gaps are significantly broadened or absent. These results offer support to the picture interpreted from the SET measurements, which suggest a gap at the CNP only in Area 1. However, while it is possible that extrinsic effects such as larger twist angle disorder may potentially explain the weak resistive feature at the superlattice gaps and lack of a feature at $\nu = \pm 2$ in Area 1, their absence makes a full detailed comparison difficult. If only a small region of the sample, comparable to the tip size, were to change conductivity, a resistive feature in an otherwise metallic background is more difficult to pick up with MIM than the reverse, as the metallic region can still screen a significant portion of the field from the tip, possibly accounting for the discrepancy. Other potential reasons for this discrepancy might be that the missing resistive features do not cross the contrast window for MIM (see Ref. [S37]), or that the sample strain and/or twist angle may have relaxed between the two measurements as the sample was warmed up and cooled down to transfer between the two cryostats.

8. CHEMICAL POTENTIAL RANGE

Here we discuss in more detail the influence of twist angle, screening, and strain on the overall chemical potential range $\Delta\mu = \mu(\nu = +4) - \mu(\nu = -4)$ spanned by the central bands and the calculations that produced Fig. 4d. We consider the range of μ between the onset of the superlattice gaps to the remote bands. Assuming that the remote bands are inert, full or empty flat band fillings should correspond to symmetry-preserving band insulators. However, $\Delta\mu$ is not simply given by the non-interacting bandwidth because interactions renormalize the band structure in a momentum- and filling-dependent manner [S38, S39]. In the experiment, the relative permittivity ϵ_r and gate-screening length $d_{\rm sc}$ are likely unchanged between different areas of the same sample, so any differences should arise from θ and strain (which is not directly probed).

A straightforward estimate of $\Delta\mu$ can obtained in mean-field theory by calculating the energy of the lowest unoccupied (highest occupied) state for the HF Hamiltonian at empty (full) filling of the central bands. There are two main subtleties to do with 'subtraction' and screening. There is no consensus on the way interactions should be combined with the BM model—the resulting ambiguity manifests as different choices in the effective single-particle kinetic energies, which evidently affects $\Delta\mu$. Furthermore in HF, no electronic screening of the gate-screened Coulomb interaction is taken into account. Regardless of these shortcomings which will impact quantitative accuracy, the overall trends with θ and strain should be captured well.

The naïve expectation is that if the moiré potential is sufficiently weak, then the only role of the twist angle would be to change the physical electronic density that corresponds to empty/full filling of the central bands. For smaller

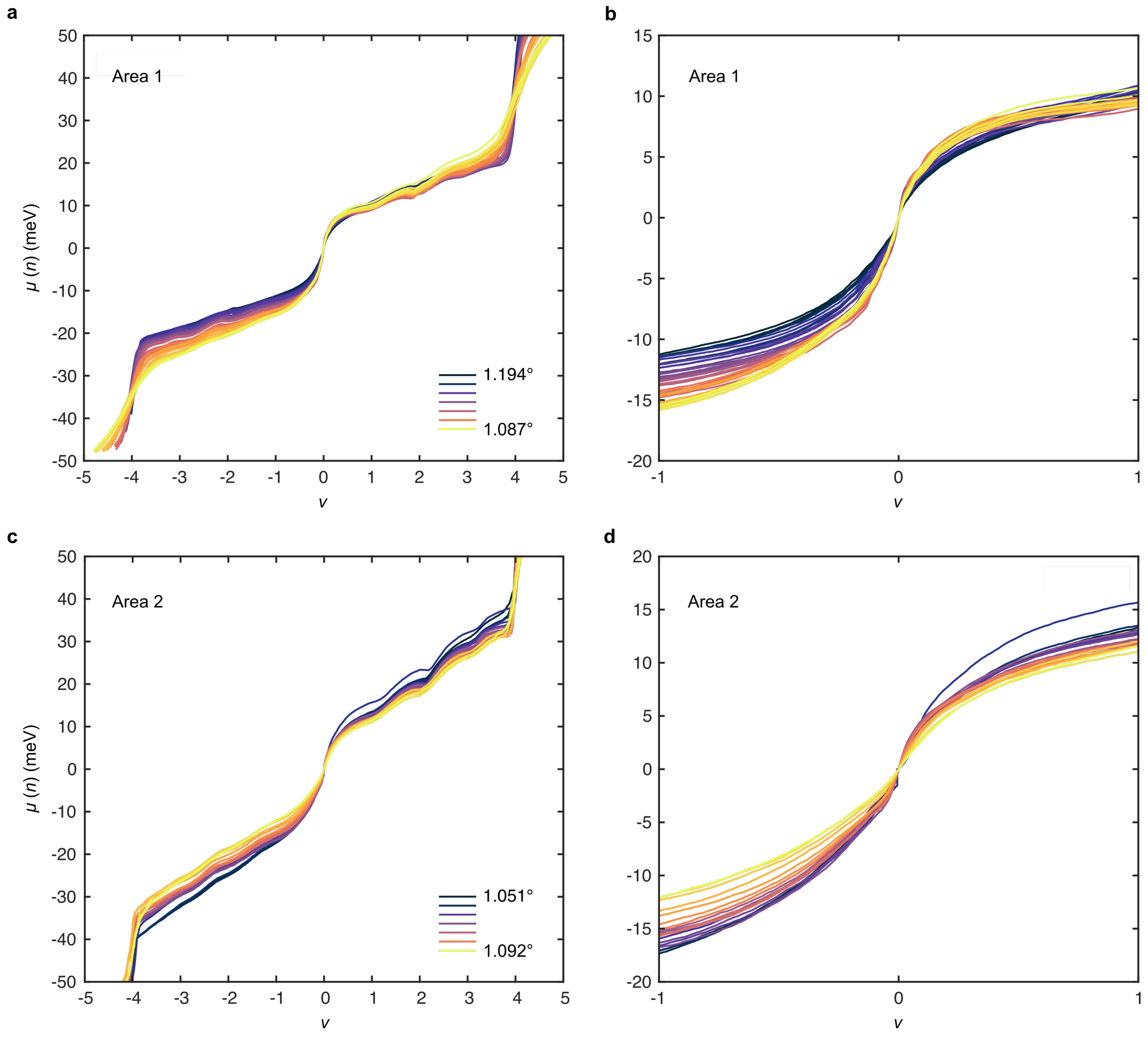

FIG. S10: **Chemical potential as a function of position. a,** Chemical potential as a function of filling factor $\mu(\nu)$ for each position along the spatial line cut in Fig. 1a (Area 1). **b,** Zoom-in of the $\mu(\nu)$ near $\nu = 0$ in (a). The slope in the vicinity of $\nu = 0$ is larger for smaller θ, in contrast to the opposite trend of the total $\Delta\mu$. **c,** $\mu(\nu)$ for each position along a spatial line cut in Area 2. **d,** Zoom-in of the $\mu(\nu)$ near $\nu = 0$ in (c).

angles, this occurs at lower densities due to the larger moiré unit cell size. If we assume that μ is a monotonically increasing function of density, then $\Delta\mu$ would be smaller for smaller angles. More simply, the interaction energy scale $\sim 1/a_M$ decreases at smaller angles. This is opposite from the behavior shown in Fig. 4c of the main text, where $\Delta\mu$ increases from around 40 meV to 80 meV when the twist angle is reduced from 1.20° to 1.05°.

Fig. S12 shows that the non-interacting BM bandwidth is not a good predictor of $\Delta\mu$. The bandwidth is too small, and has a pronouced minimum near the magic angle, a feature which is robust for different values of $w_{\rm AA}$ and finite strain. Adding finite interaction strength, we recover the correct overall trend of $\Delta\mu$ seen experimentally. This trend is robust to different choices of $w_{\rm AA}$ and subtraction schemes. In the 'graphene scheme', the subtracted P_0 corresponds to the density matrix of decoupled graphene layers [S10, S11].

The rightmost two columns in Fig. S12 were generated for a fixed $\epsilon_r = 6$—a weaker interaction (and hence larger ϵ_r) would decrease $\Delta\mu$. In addition to reducing twist angle, increasing heterostrain and $w_{\rm AA}$ also increases $\Delta\mu$. Since only the twist angle is directly measured in experiment, and given the interaction scheme dependence, it is not possible to use $\Delta\mu$ to quantitatively determine $w_{\rm AA}, \epsilon_r$, and the local values of strain. However, the large experimentally observed variation in $\Delta\mu$ in the range $\theta = 1.05° - 1.20°$ suggests the presence of greater heterostrain in the sample region of smaller twist angle.

We also comment on the magnetic field dependence of the chemical potential and its relationship with magnetic

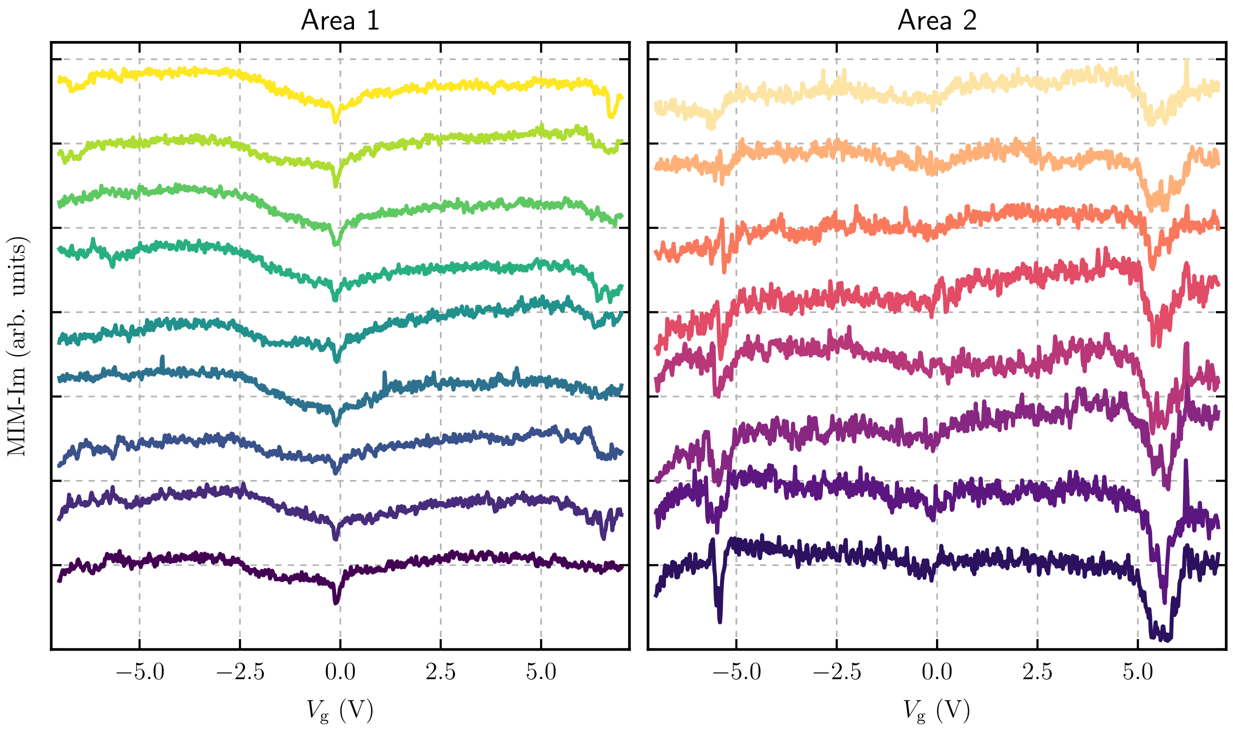

FIG. S11: **Microwave Impedance Microscopy (MIM) measurements in Area 1 and Area 2 at zero magnetic field.** MIM-Im signal as a function of gate voltage V_g measured at different points on a grid covering 300 nm × 300 nm over the regions Area 1 and Area 2 identified with SET.

moments. Fig. S13 shows the analogous plot of μ with increasing B in the vicinity of $\nu = 2$. In [S26, S42], the magnetic moment per electron was obtained by invoking the Maxwell relation: $\partial\mu/\partial B = -\partial m/\partial\nu$. It is therefore tempting to consider applying the same method to the data presented in Fig. S13. However, when applying a perpendicular magnetic field, this method is complicated by orbital effects, such as the formation and deformation of Hofstadter subbands/Landau levels. As can be seen from Fig. S13, the magnetic field dramatically shifts the chemical potential throughout the full range of fillings, not just near $\nu = 2$. The decrease in $\Delta\mu_{tot} = \mu(\nu = 4) - \mu(\nu = -4)$ with increasing B is consistent with the expectations from single-particle and interaction-renormalized Hofstadter spectrum calculations [S9, S31, S32, S40, S41, S43]. This effect will complicate extraction of the magnetic moment, as one would naively obtain a large ($\sim 7\mu_B$ per unit cell) magnetic moment, but it cannot be unambiguously attributed solely to the large spin quantum number of the skyrmionic excitations.

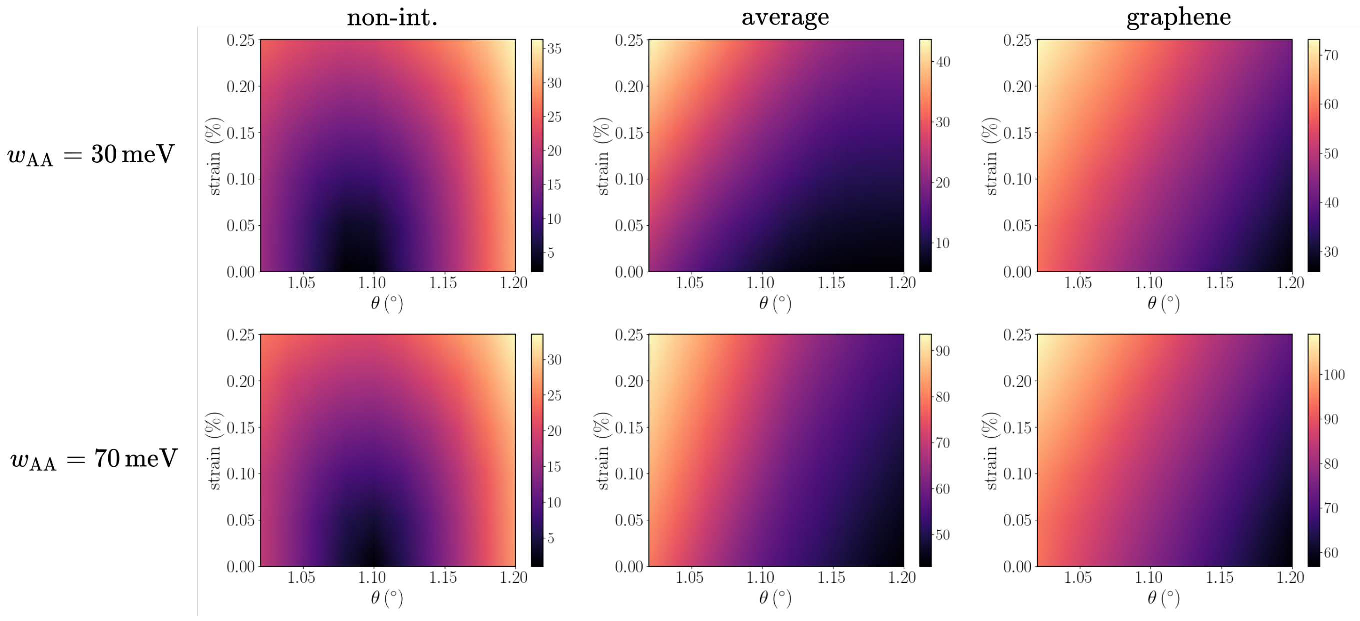

FIG. S12: **Numerical computation for the chemical potential range $\Delta\mu = \mu(\nu = +4) - \mu(\nu = -4)$.** $\Delta\mu$ is computed for two values of the chiral ratio and three interaction schemes (non-int. refers to the bare BM model bandwidth). Interactions are single-gate screened with $d_{\rm sc} = 30\,$nm and $\epsilon_r = 6$.

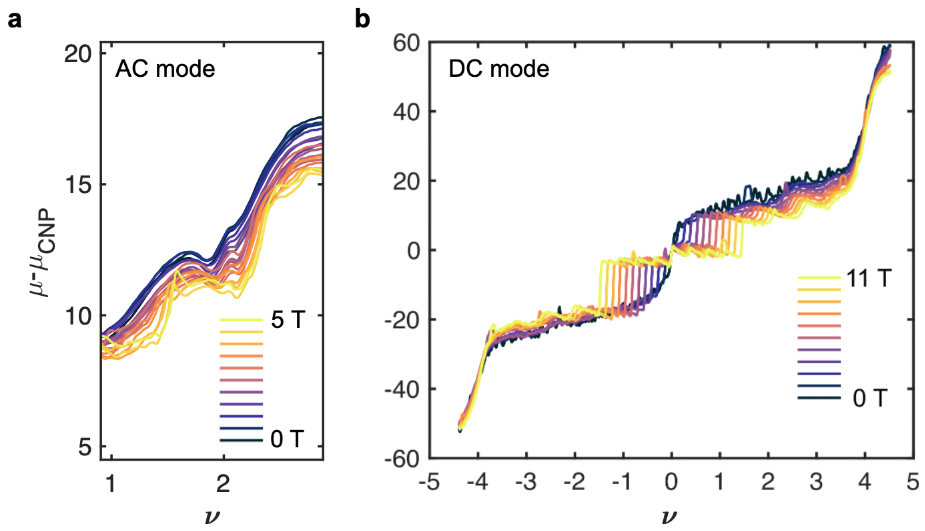

FIG. S13: **Chemical potential traces at different magnetic fields at $\theta = 1.14°$. a,** Chemical potential relative to the CNP in the vicinity of $\nu = 2$. A monotonic trend of decreasing μ with increasing B can be seen. **b,** Chemical potential in the entire filling range. Decreasing $\Delta\mu_{\rm tot} = \mu(\nu = +4) - \mu(\nu = -4)$ is likely a result of the band deformation in the Hofstadter regime [S8, S9, S31, S32, S40, S41]. In (b), we have used a different method (DC mode, see [S8] for details) to obtain the chemical potential; this is necessary due to spurious enhancement when using the AC method from large sample/contact resistance over certain ranges of filling factor.

[S1] Y. Choi, H. Kim, Y. Peng, A. Thomson, C. Lewandowski, R. Polski, Y. Zhang, H. S. Arora, K. Watanabe, T. Taniguchi, J. Alicea, and S. Nadj-Perge, Nature **589**, 536 (2021).
[S2] J. M. Park, Y. Cao, K. Watanabe, T. Taniguchi, and P. Jarillo-Herrero, Nature **592**, 43 (2021).
[S3] Y. Saito, J. Ge, L. Rademaker, K. Watanabe, T. Taniguchi, D. A. Abanin, and A. F. Young, Nature Physics **17**, 478 (2021).
[S4] S. Tomarken, Y. Cao, A. Demir, K. Watanabe, T. Taniguchi, P. Jarillo-Herrero, and R. Ashoori, Physical Review Letters **123**, 046601 (2019).
[S5] K. P. Nuckolls, M. Oh, D. Wong, B. Lian, K. Watanabe, T. Taniguchi, B. A. Bernevig, and A. Yazdani, Nature **588**, 610 (2020).
[S6] I. Das, X. Lu, J. Herzog-Arbeitman, Z.-D. Song, K. Watanabe, T. Taniguchi, B. A. Bernevig, and D. K. Efetov, Nature Physics (2021), 10.1038/s41567-021-01186-3.
[S7] S. Wu, Z. Zhang, K. Watanabe, T. Taniguchi, and E. Y. Andrei, Nature Materials **20**, 488 (2021).
[S8] J. Yu, B. A. Foutty, Z. Han, M. E. Barber, Y. Schattner, K. Watanabe, T. Taniguchi, P. Phillips, Z.-X. Shen, S. A. Kivelson, and B. E. Feldman, Nature Physics , 1 (2022), publisher: Nature Publishing Group.
[S9] R. Bistritzer and A. H. MacDonald, Physical Review B **84**, 035440 (2011).
[S10] M. Xie and A. MacDonald, Physical Review Letters **124**, 097601 (2020).
[S11] N. Bultinck, E. Khalaf, S. Liu, S. Chatterjee, A. Vishwanath, and M. P. Zaletel, Physical Review X **10**, 031034 (2020).
[S12] Y. Kwan, G. Wagner, T. Soejima, M. Zaletel, S. Simon, S. Parameswaran, and N. Bultinck, Physical Review X **11**, 041063 (2021), publisher: American Physical Society.
[S13] A. T. Pierce, Y. Xie, J. M. Park, E. Khalaf, S. H. Lee, Y. Cao, D. E. Parker, P. R. Forrester, S. Chen, K. Watanabe, T. Taniguchi, A. Vishwanath, P. Jarillo-Herrero, and A. Yacoby, Nature Physics **17**, 1210 (2021), number: 11 Publisher: Nature Publishing Group.
[S14] M. Serlin, C. L. Tschirhart, H. Polshyn, Y. Zhang, J. Zhu, K. Watanabe, T. Taniguchi, L. Balents, and A. F. Young, Science **367**, 900 (2020).
[S15] Y. H. Kwan, G. Wagner, N. Bultinck, S. H. Simon, and S. A. Parameswaran, arXiv:2112.06936 [cond-mat] (2021), arXiv: 2112.06936.
[S16] E. Khalaf and A. Vishwanath, arXiv:2112.06935 [cond-mat] (2021), arXiv: 2112.06935.
[S17] F. Schindler, O. Vafek, and B. A. Bernevig, Physical Review B **105**, 155135 (2022), publisher: American Physical Society.
[S18] P. J. Ledwith, E. Khalaf, Z. Zhu, S. Carr, E. Kaxiras, and A. Vishwanath, (2021), 10.48550/arXiv.2111.11060.
[S19] O. Vafek and J. Kang, Physical Review Letters **125**, 257602 (2020).
[S20] S. Chatterjee, N. Bultinck, and M. P. Zaletel, Physical Review B **101**, 165141 (2020), publisher: American Physical Society.
[S21] N. Bultinck, S. Chatterjee, and M. P. Zaletel, Physical Review Letters **124**, 166601 (2020).
[S22] E. Khalaf, S. Chatterjee, N. Bultinck, M. P. Zaletel, and A. Vishwanath, Science Advances **7**, eabf5299 (2021), publisher: American Association for the Advancement of Science.
[S23] E. Khalaf, N. Bultinck, A. Vishwanath, and M. P. Zaletel, (2020), 10.48550/arXiv.2009.14827.
[S24] B. Douçot, M. O. Goerbig, P. Lederer, and R. Moessner, Physical Review B **78**, 195327 (2008).
[S25] B. Lian, Z.-D. Song, N. Regnault, D. K. Efetov, A. Yazdani, and B. A. Bernevig, Physical Review B **103**, 205414 (2021).
[S26] Y. Saito, F. Yang, J. Ge, X. Liu, T. Taniguchi, K. Watanabe, J. I. A. Li, E. Berg, and A. F. Young, Nature **592**, 220 (2021).
[S27] M. Yankowitz, S. Chen, H. Polshyn, Y. Zhang, K. Watanabe, T. Taniguchi, D. Graf, A. F. Young, and C. R. Dean, Science **363**, 1059 (2019).
[S28] Y. Cao, V. Fatemi, A. Demir, S. Fang, S. L. Tomarken, J. Y. Luo, J. D. Sanchez-Yamagishi, K. Watanabe, T. Taniguchi, E. Kaxiras, R. C. Ashoori, and P. Jarillo-Herrero, Nature **556**, 80 (2018).
[S29] D. Xiao, J. Shi, and Q. Niu, Physical Review Letters **95**, 137204 (2005).
[S30] T. Thonhauser, D. Ceresoli, D. Vanderbilt, and R. Resta, Physical Review Letters **95**, 137205 (2005), publisher: American Physical Society.
[S31] D. Parker, P. Ledwith, E. Khalaf, T. Soejima, J. Hauschild, Y. Xie, A. Pierce, M. P. Zaletel, A. Yacoby, and A. Vishwanath, "Field-tuned and zero-field fractional Chern insulators in magic angle graphene," (2021), arXiv:2112.13837 [cond-mat].
[S32] X. Wang and O. Vafek, arXiv:2112.08620 [cond-mat] (2022), arXiv: 2112.08620.
[S33] O. Antebi, A. Stern, and E. Berg, Physical Review B **105**, 104423 (2022).
[S34] Z. Bi, N. F. Q. Yuan, and L. Fu, Physical Review B **100**, 035448 (2019).
[S35] D. E. Parker, T. Soejima, J. Hauschild, M. P. Zaletel, and N. Bultinck, Physical Review Letters **127**, 027601 (2021), publisher: American Physical Society.
[S36] Y. Choi, H. Kim, C. Lewandowski, Y. Peng, A. Thomson, R. Polski, Y. Zhang, K. Watanabe, T. Taniguchi, J. Alicea, and S. Nadj-Perge, arXiv:2102.02209 [cond-mat] (2021), arXiv: 2102.02209.
[S37] M. E. Barber, E. Y. Ma, and Z.-X. Shen, Nature Reviews Physics **4**, 61 (2022).
[S38] J. Kang, B. A. Bernevig, and O. Vafek, Physical Review Letters **127**, 266402 (2021), publisher: American Physical Society.
[S39] F. Guinea and N. R. Walet, Proceedings of the National Academy of Sciences **115**, 13174 (2018), publisher: Proceedings

of the National Academy of Sciences.
[S40] P. Moon and M. Koshino, Physical Review B **85**, 195458 (2012).
[S41] K. Hejazi, C. Liu, and L. Balents, Physical Review B **100**, 035115 (2019).
[S42] U. Zondiner, A. Rozen, D. Rodan-Legrain, Y. Cao, R. Queiroz, T. Taniguchi, K. Watanabe, Y. Oreg, F. von Oppen, A. Stern, E. Berg, P. Jarillo-Herrero, and S. Ilani, Nature **582**, 203 (2020).
[S43] J. Herzog-Arbeitman, A. Chew, D. K. Efetov, and B. A. Bernevig, Physical Review Letters **129**, 076401 (2022), publisher: American Physical Society.
